# miR-4270 suppresses hepatocellular carcinoma progression by inhibiting DNMT3A-mediated methylation of HGFAC promoter

**DOI:** 10.7717/peerj.16566

**Published:** 2023-12-05

**Authors:** Qiang Zou, Shasha Cao

**Affiliations:** 1 Department of Interventional Therapy, Tianjin Medical University Cancer Institute and Hospital, National Clinical Research Center for Cancer, Key Laboratory of Cancer Prevention and Therapy, Tianjin’s Clinical Research Center for Cancer, Tianjin, China; 2 Department of Neonatology, Zibo Maternal and Child Health Hospital, Zibo, China

**Keywords:** Hepatocellular carcinoma, miR-4270, DNMT3A, HGFAC

## Abstract

**Background:**

miR-4270 is a regulatory factor has been linked with the progression of various cancers, such as nasopharyngeal carcinoma, hepatocellular carcinoma (HCC), and gastric cancer. However, the underlying mechanisms through which miR-4270 modulates HCC development are not fully understood.

**Methods:**

miR-4270 expression levels were analyzed in various HCC cell lines and tissue samples. An online bioinformatics tool was then utilized to predict the miR-4270 target gene. The binding relationship between miR-4270 and its target gene *DNMT3A* was verified using dual-luciferase reporter and Ago2–RIP assays. Then, co-immunoprecipitation (Co-IP) and chromatin immunoprecipitation (ChIP) assays were conducted to investigate the association between *DNMT3A* and the hepatocyte growth factor activator (*HGFAC*) promoter region. To assess the methylation level of the *HGFAC* promoter, methylation-specific PCR (MSP) was employed. Furthermore, rescue analyses were carried out to evaluate the functional relevance of miR-4270 and *HGFAC* in the modulation of the malignant properties of HCC cells. Finally, HepG2 cells overexpressing miR-4270 were subcutaneously injected into nude mice to estimate the impact of miR-4270 on the xenograft tumor growth of HCC.

**Results:**

A substantial miR-4270 downregulation was revealed in HCC patient samples and cell lines. miR-4270 upregulation suppressed both cell proliferation and invasion while promoting apoptosis. At the molecular level, miR-4270 was found to bind to the 3′untranslated region (3′UTR) of *DNMT3A*, thereby inhibiting *DNMT3A*-mediated methylation of the *HGFAC* promoter. Functional assays indicated that inhibition of miR-4270 stimulated HCC cell growth, an effect counteracted by overexpression of *HGFAC*. *In vivo* assays further verified that miR-4270 effectively suppressed the progression of HCC xenograft tumors.

**Conclusions:**

miR-4270 was found to mitigate the malignant characteristics of HCC by inhibiting *DNMT3A*-mediated methylation of the *HGFAC* promoter, suggesting a potential therapeutic avenue for the management of HCC.

## Introduction

Liver cancer is among the leading causes of cancer-associated mortality throughout the world. Hepatocellular carcinoma (HCC) is the most prevalent type of primary liver cancer (Shen et al., 2022; Ong, Huey & Shelat, 2022). The incidence of HCC is highest in Asia and Africa, primarily due to prevalent underlying conditions such as liver disease and cirrhosis. Infections by the hepatitis B and C viruses are the most common causes of HCC, followed by cirrhosis (Xu et al., 2019). Additional risk factors for liver cancer include alcohol abuse, obesity, excess iron, environmental pollutants, and exposure to aflatoxins. Surgical resection stands as the primary treatment modality for HCC at present. Nonetheless, the high propensity for metastasis and recurrence in HCC often undermines post-surgical prognosis, with the accompanying surgical trauma presenting further complications (Piñero, Dirchwolf & Pessôa, 2020). With advancements such as the completion of human genome sequencing, therapies targeting specific molecular pathways are emerging as breakthrough approaches. Therefore, identifying target genes that can suppress HCC cell migration and invasion has become an important research direction in this field.

MicroRNAs (miRNAs) are approximately endogenous RNAs between 19 and 25 base pairs in length. They are non-coding and interact primarily with the 3′ untranslated regions (3′UTRs) of target genes, resulting in RNA degradation or the suppression of translation, and thus post-transcriptionally downregulating gene expression (Lu & Rothenberg, 2018). More than 2,500 miRNAs have been discovered to date, each capable of affecting the expression of hundreds of genes. MiRNAs are essentially associated with various cellular biological mechanisms, such as development, differentiation, growth, and apoptosis (Ni & Leng, 2016; Wang et al., 2022b). Therefore, changes in miRNA expression levels can disrupt these cellular functions, potentially leading to various diseases. Decades of research focusing on the qualitative and quantitative evaluation of miRNA levels have revealed significant alterations in their expression profiles across different diseases (Oura, Morishita & Masaki, 2020; Yan et al., 2022b). These findings underscore the potential of miRNA expression profiling as a valuable tool in disease diagnosis and cure. Some miRNAs are highly expressed in HCC and function as oncogenes, while others are under-expressed, serving as tumor suppressors (Wong, Tsang & Ng, 2018; Komoll et al., 2021). For instance, decreased miR-744-5p expression has been observed in HCC tissues and cells, whereas its upregulation significantly reduced HCC cell and tissue growth (Huang et al., 2021; Yu et al., 2022). Research studies have indicated the downregulation of miR-515-5p in HCC tissues, with its upregulation significantly suppressing malignant behaviors in HCC cells (Ni et al., 2020). Dong et al. (2021) discovered that downregulated expression of miR-369 in HCC tissues predicted poor prognosis in patients with HCC (Dong et al., 2021; Wang et al., 2022a). miR-4270, a recently identified miRNA, is under-expressed in different cancers, such as nasopharyngeal carcinoma, retinoblastoma, and gastric cancer, where it acts as a tumor suppressor influencing processes such as tumor growth, invasion, and metastasis (Hao et al., 2021; Feng et al., 2021; Shen et al., 2020). Nevertheless, there are few reports on the relationship between HCC and miR-4270. Our analysis revealed the downregulation of miR-4270 in HCC clinical tissue samples within the GEO datasets, indicating a need for comprehensive investigation into its functional roles and molecular mechanisms in HCC.

Epigenetic modifications, encompassing RNA and non-coding RNA modifications, histone modifications, DNA methylation, and chromatin remodeling, are crucial for regulating gene expression and maintaining cellular identity. Numerous studies have shown that abnormal epigenetic reprogramming is a key driver in the pathogenesis of various human malignant tumors. DNA methylation is a typical form of epigenetic regulation, that is primarily catalyzed by DNA methyltransferases (DNMTs), including *DNMT3A*, *DNMT1*, and *DNMT3B* (Long et al., 2019; Liu et al., 2022; Cheng et al., 2018). Methylation typically occurs within CpG islands in the promoter regions of genes, often leading to durable and heritable gene silencing. CpG islands, rich in CpG sequences and ranging from 200 to several thousand base pairs, are predominantly situated near gene promoters. It has been suggested that abnormal promoter DNA methylation is related to tumor occurrence. *DNMT3A* has a molecular weight of 130 kDa and is localized on the human 2p23 chromosome. In mammals, the protein is significantly conserved with 98% homology between humans and mice. Numerous studies have reported that aberrant overexpression of *DNMT3A* contributes to the progression of various cancers, including colorectal cancer (Zhou et al., 2022), ovarian cancer (He et al., 2019), pancreatic ductal adenocarcinoma (Jing et al., 2019), and breast cancer (Man et al., 2022). While the precise mechanisms behind aberrant DNA methylation remain unclear, multiple studies have shown a significant upregulation of *DNMT3A* mRNA levels in cirrhotic tissues in comparison with normal hepatic tissues, with even increased expression in primary HCC tissues compared to surrounding non-tumorous tissues (Wang et al., 2021).

Based on these insights, our study employed both *in vivo* and *in vitro* analyses to elucidate the effects of miR-4270 on the biological behavior of HCC cells. Bioinformatics was then utilized to predict and analyze the potential relationship and underlying mechanisms between miR-4270 and *DNMT3A*, offering a novel perspective for gene therapy approaches in HCC.

## Materials and Methods

### HCC tissue specimens

Data were collected from 114 HCC patients treated at our hospital between October 2017 and January 2020, comprising 35 females and 79 males between the ages of 31 and 78 years (average age = 48.55 ± 10.17 years). Inclusion parameters were patients with confirmed diagnosis through surgical pathology, imaging, and other relevant examinations, complete admission data, no history of prior antitumor treatments, aged over 20 years, and who had consented to laparoscopic radical hepatectomy for HCC. The exclusion criteria were the presence of neurological diseases, other malignant tumors, a combination of secondary HCC or liver abscess hemangioma, abnormal function of the heart, liver, kidney, and other important organs, inability to communicate normally, involvement in other clinical studies, or withdrawal from the present study. This investigation was authorized by the Ethics Committee of Tianjin Medical University Cancer Hospital, and all the participants provided written informed consent.

### Bioinformatics assay

The GEO database (https://www.ncbi.nlm.nih.gov/geo/) was accessed, and a search using ‘hepatocellular carcinoma’ as the keywords was conducted. ‘*Homo sapiens*’ and ‘expression profiling by array’ were selected, and the gene data matrix files for GSE108724 were downloaded. The GSE108724 microarray files, sourced from the GPL20712 platform (HuGene-1_0-st Affymetrix Human Gene 1.0 ST Array (transcript (gene) version), comprised 7 HCC samples and seven corresponding adjacent non-tumor tissue samples. All microarray data were processed for batch effects with the support of the R package “sva.” Following batch effect processing, we further standardized the data. The normalization process encompassed three steps: (1) correcting the background data, which involved cleaning unbound probe impurities to detect genes with low differential expression; (2) conducting normalization to remove systematic measurement errors, ensuring comparability of measurements across experimental conditions; (3) utilizing robust array average algorithm processing to derive consistent average fluorescence intensity values from the probe group, preparing the GEO data for subsequent comparative analyses. Differentially expressed genes (DEGs) were determined through differential analysis using the ‘Limma’ package in R software. The GEPIA database (http://gepia.cancer-pku.cn/) was utilized to assess *DNMT3A* and *HGFAC* expression levels in 369 HCC tumor samples and 160 paired normal tissues spanning various stages of HCC. Additionally, the correlations between the levels of *DNMT3A* and *HGFAC* and patient outcomes, specifically overall survival (OS) and disease-free survival (DFS), were evaluated.

### Cell lines and culture

The HCC cell lines SMMC-7721, Hep3B, MHCC97H, HepG2, and Huh7, and the human normal hepatocyte L02 cell line, were cultured in DMEM containing penicillin-streptomycin (1%; Sigma-Aldrich, St. Louis, MI, USA) and 10% fetal bovine serum (FBS; Thermo Fisher, Waltham, MA, USA) in a 5% CO_2_ incubator at 37 °C. Cells were grown until 80% confluent before detachment with trypsin and passaging at a ratio of 1:2 to 1:3.

### Cell transfection

HCC cells in logarithmic growth were plated at 2 × 10^3^cells/well in 96-well plates. An miR-4270 mimic, miR-4270 inhibitor, and pcDNA-*HGFAC* (GenePharma Co., Ltd., Shanghai, China) were transfected into cells at 70% confluence using Lipofectamine 3000, according to the provided protocol. After 48 h, the cells were harvested for subsequent analyses.

### RT-qPCR

Total RNA was extracted from HCC tissues and cells using TRIzol reagent (Invitrogen, Carlsbad, CA, USA). The reverse transcription reaction (20 μL) comprised 10 μL total RNA, 1.2 μL miR-RT primer (1 μmol/L), MMLV (0.2 μL, 200 U/μL), 5 × RT buffer (4 μL), dNTP (0.75 μL, 10 mmol/L), and RNase-free ddH_2_O (3.85 μL). We assessed the levels of miR-4270, *DNMT3A*, and *HGFAC* in HCC tissues and cells using the miScript PCR starter kit (Qiagen, Hilden, Germany) and amplified the mRNA on a Bio-Rad CFX90 Real-time PCR system. The 2^−ΔΔCT^ method was utilized for assessing the relative expression levels. The primers for miR-4270, *DNMT3A*, and *HGFAC* used in this investigation were as follows: miR-4270 forward, 5′-GCC GAG TCA GGG AGT CAG GG-3′; and reverse, 5′-CTC AAC TGG TGT CGT GGA-3′; *DNMT3A* forward, 5’-GTG GAT GTT GAT GGG AGG CA-3′; and reverse, 5′-CGC ACC ACT GTT TTC ACC AG-3′; *HGFAC* forward: 5′-AGG GAG CTA GAA AGA GGG GG-3′; and reverse, 5′-AGG CTC CAG GGG TCT CTT AG-3′.

### Western blotting

Briefly, proteins were separated on SDS-PAGE gels comprising 10% separating gel and 5% concentrating gel. The proteins were then transferred to PVDF membranes according to the molecular weights of the target proteins after electrophoresis. After electrotransfer, 5% BSA was used to block the membranes for 3 h at room temperature. The membrane was then incubated on a shaker at 4 °C in diluted primary antibodies against DNMT3A and HGFAC overnight. The membranes were then washed three times with 1 × TBST, each wash lasting 10 min, and were then incubated in a diluted secondary antibody (goat anti-rabbit IgG) solution for 1 h at ambient temperature and washed three times with 1 × TBST, with each wash lasting 10 min. Finally, the luminescent substrate was prepared per the reagent instructions and applied to the PVDF membrane, and the membrane was observed using an automatic imager.

### Cell proliferation assay

HCC cells (1 × 10^3^/mL) were seeded into 96-well plates in 100 μL cell suspension/well. After 24, 48, and 72 h, CCK-8 solution (10 μL) was added to the cells for 1 h. The absorbances (OD values) at 450 nm were measured using a microplate reader.

### Cell invasion assay

Following the Transwell chamber protocol, 200 μl of each differently transfected HCC cell suspension (serum-free) were placed in the Matrigel-coated upper chamber while 500 μl of DMEM containing 10% FBS was added to the lower chamber. After 24 h, the cells were stained with crystal violet and visualized by microscopy (IX53; Olympus Corp, Tokyo, Japan) and counted. Each assay was performed three times.

### Cell cycle assay

Exponentially growing cells were seeded in six-well plates and then processed and collected using a Cell Cycle Staining kit, following standardized procedures. Subsequently, cell cycle distribution was evaluated by flow cytometry and assessed *via* the FlowJo 7.6 software.

### Cell apoptosis assay

HCC cells from each treatment group were collected and centrifuged at 2,000 rpm for 5 min, followed by three rinses with PBS. Subsequently, the cells were resuspended in binding buffer (500 μL). Then propidium iodide (PI) and Annexin V/FITC (Pricella, Wuhan, China) were added (5 μL, respectively). After incubation for 15 min in the dark, a FACScan flow cytometer (Attune NxT; Thermo Fisher, Waltham, MA, USA) was used to analyze the proportions of apoptotic cells with Annexin V or PI-stained cells identified as apoptotic cells.

### Methylation-specific PCR

DNA bisulfite modification relies on the ability of bisulfite and hydroquinone to convert cytosine into uracil on a DNA strand, a reaction prevented by cytosine methylation. In this way, methylated *vs* unmethylated DNA sequences can be distinguished by sequence-specific primers after bisulfite modification. Here, 1‒2 μL of genomic DNA was diluted to 30 μL and denatured by adding 20 μL 0.5 mol/L NaOH at 37 °C. Then, 520 μL of freshly prepared 3 mol/L NaHSO_3_ (pH 5.0) and 30 μL of 10 mmol/L hydroquinone were added, mixed, and incubated for 16 h at 50 °C. The sodium bisulfite-modified DNA was recovered and purified by dialysis against 1% agarose, and then 50 μL of 0.6 mol/L NaOH was added at 37 °C to terminate the reaction. DNA was recovered by ethanol precipitation, dissolved in 30 μL sterile double-distilled water. Finally, the collected DNA was used immediately for PCR amplification or stored at −80 °C.

### Co-immunoprecipitation (Co-IP)

The Pierce™ Direct IP Kit 26148 was used, following the manufacturers’ guide (Takara Biotechnology, Dalian, China). Twenty microliters of Amino Link PlusResin and Pierce Control Agarose Resin (a negative control to eliminate nonspecific protein binding) were added with 4 μg of an anti-HGFAC rabbit monoclonal antibody for 100 min. Subsequently, the protein sample (500 μL) was added and incubated overnight, followed by four rinses with IP lysis/wash buffer and one with conditioning buffer. HGFAC protein complexes were then eluted with solution buffer, and 10% SDS-PAGE electrophoresis (voltage 110 V, electrophoresis time 25 min) was performed, followed by western blotting, as described above. The membranes were incubated with rabbit anti-human HGFAC (1:1,000 dilution) and DNMT3A (1:1,000 dilution) primary antibodies overnight at 4 °C. After three washes in TBST, the membranes were developed using an enhanced chemiluminescence (ECL) reagent.

### Chromatin immunoprecipitation (ChIP)

HCC cells from each group were fixed with 1% formaldehyde for 10 min, and the membranes and nuclei were sequentially lysed. Chromatin DNA was fragmented ultrasonically into fragments between 200 and 1,000 BP. The chromatin suspension was diluted five-fold with dilution buffer, and 5% was reserved as control (input). We then aliquoted the remainder, adding an anti-DNMT3A antibody to one portion and a normal rabbit IgG antibody to another, incubating on a shaker overnight at 4 °C. Protein A/G magnetic beads were utilized for collection of the immunoprecipitated complexes, followed by extensive washing in a 65 °C water bath overnight. DNA was extracted using a PCR product extraction kit (Qiagen, Hilden, Germany) with the total DNA (input) and each immunoprecipitated DNA was used as a template. The results were calculated as the relative expression level (% input) using the following formula:

$relative\; expression\; level\; = \; 2\;\sim \; \left[ {CT\; \left( {input} \right)\; - \; CT\; \left( {chip} \right)} \right]\; \times\; dilution\; factor\;$

This represents the ratio of immunoprecipitated DNA to total DNA (input). The relative expression level (% input) of rabbit normal IgG precipitated DNA *vs* input DNA was taken as a negative control.

### Xenograft tumor mouse models

BALB/c-nu nude mice (age = 4 weeks, male, *n* = 24) were allowed to acclimatize for 2 days in a specific pathogen-free (SPF) facility. HepG2 cells (5 × 10^6^ in a 200 μL suspension) were inoculated into the right groin area of the mice, after which the animals were randomly divided into the NC mimic and miR-4270 mimic groups (each *n* = 12). The tumor growth was monitored and the long and short diameters of the tumors were measured every 5 days. The tumor volumes were calculated using the formula:

$Tumor\; volume = \displaystyle{1 \over 2} \times long\; diameter \times short\; diamete{r^2}$

The mice were euthanized after 30 days and the tumors were harvested and stored for later use.

### Statistical measurements

Statistical analyses were conducted using GraphPad Prism 7.0 software. Three replicates were used for all assays. Data distributions were verified using the Shapiro-Wilk test, and the homogeneity of variances was verified by Levene’s test. Normally distributed and variance-homogenous data were analyzed by one-way analysis of variance (ANOVA) or t-tests followed by *post hoc* analysis (Tukey-Kramer correction). Non-normally distributed or non-homogenous data were analyzed using Kruskal-Wallis non-parametric tests. *P* < 0.05 was considered statistically significant.

## Results

### miR-4270 levels were downregulated in HCC tissues and cell lines

**Figure 1:**
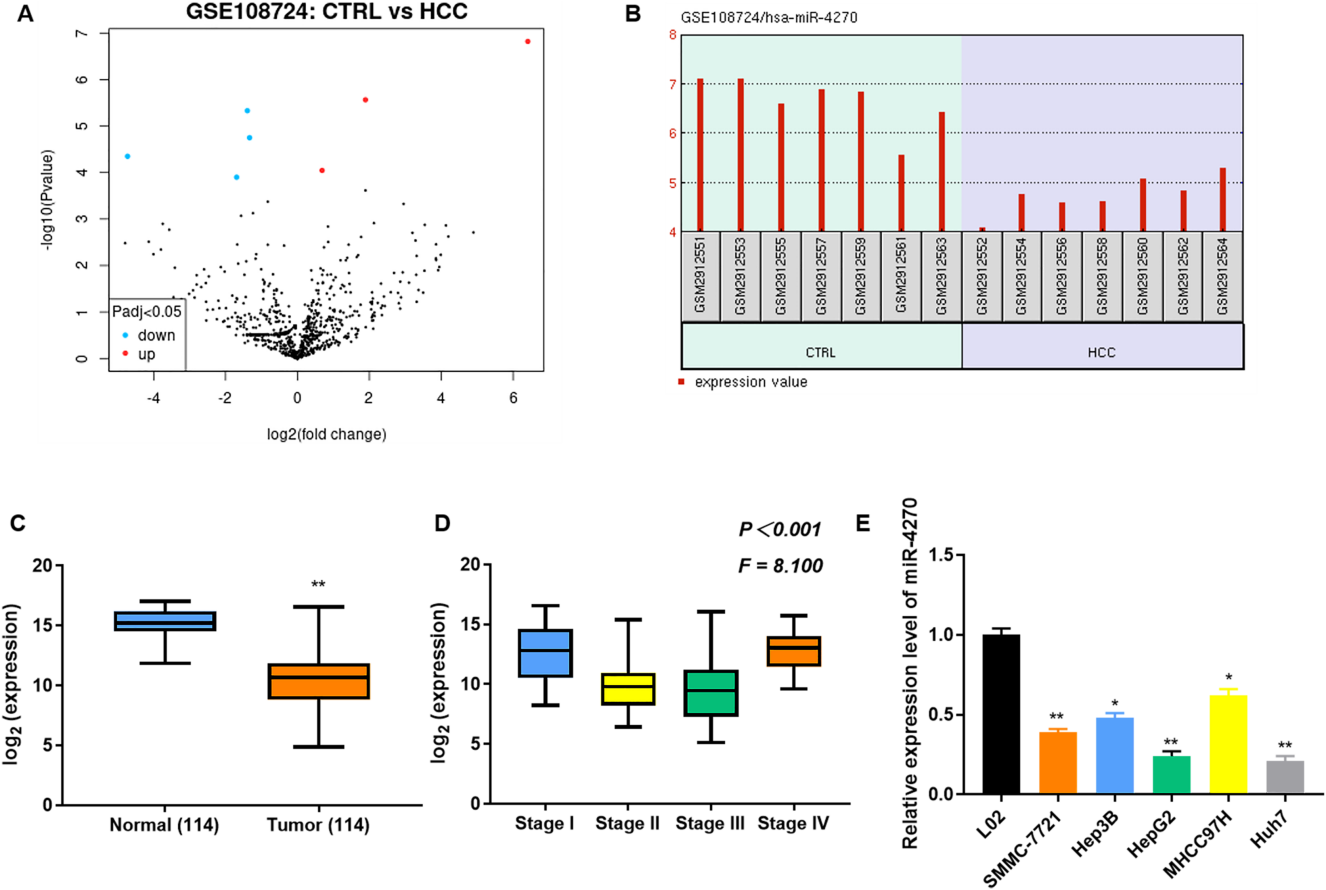
Downregulation of miR-4270 in HCC tissues and cell lines. (A) Volcano plot illustrating the differential expression of genes in the GSE108724 dataset, comprising 7 HCC and seven adjacent non-tumor tissue samples. The x-axis represents the log2 (fold change). (B) Expression levels of miR-4270 in individual samples from the GSE108724 dataset. (C) Comparative expression of miR-4270 in 114 paired HCC tumor and adjacent non-tumor tissues. **P < 0.01 *vs* Normal, non-paired *t*-test. (D) miR-4270 expression analysis across different HCC clinical stages, one-way ANOVA, *P* < 0.001, F = 8.100. (E) miR-4270 levels in different HCC cell lines. *P < 0.05, **P < 0.01 *vs* L02. *N* = 6.

Seven differentially expressed miRNAs (three upregulated and four downregulated) were identified in the GSE108724 dataset (Fig. 1A). Among them, miR-4270 showed significant downregulation in HCC tissues compared with normal tissues (Fig. 1B). Furthermore, to confirm the expression of miR-4270 in HCC, the miR-4270 levels in HCC and normal tissues at different stages, as well as in the HCC cell lines (SMMC-7721, Hep3B, HepG2, MHCC97H, and Huh7) were measured *via* RT-qPCR. The results indicated that miR-4270 was markedly downregulated in HCC, and its expression correlated with various clinical stages of HCC (Figs. 1C and 1E).

### miR-4270 mimic repressed HCC cell proliferation and invasion

**Figure 2:**
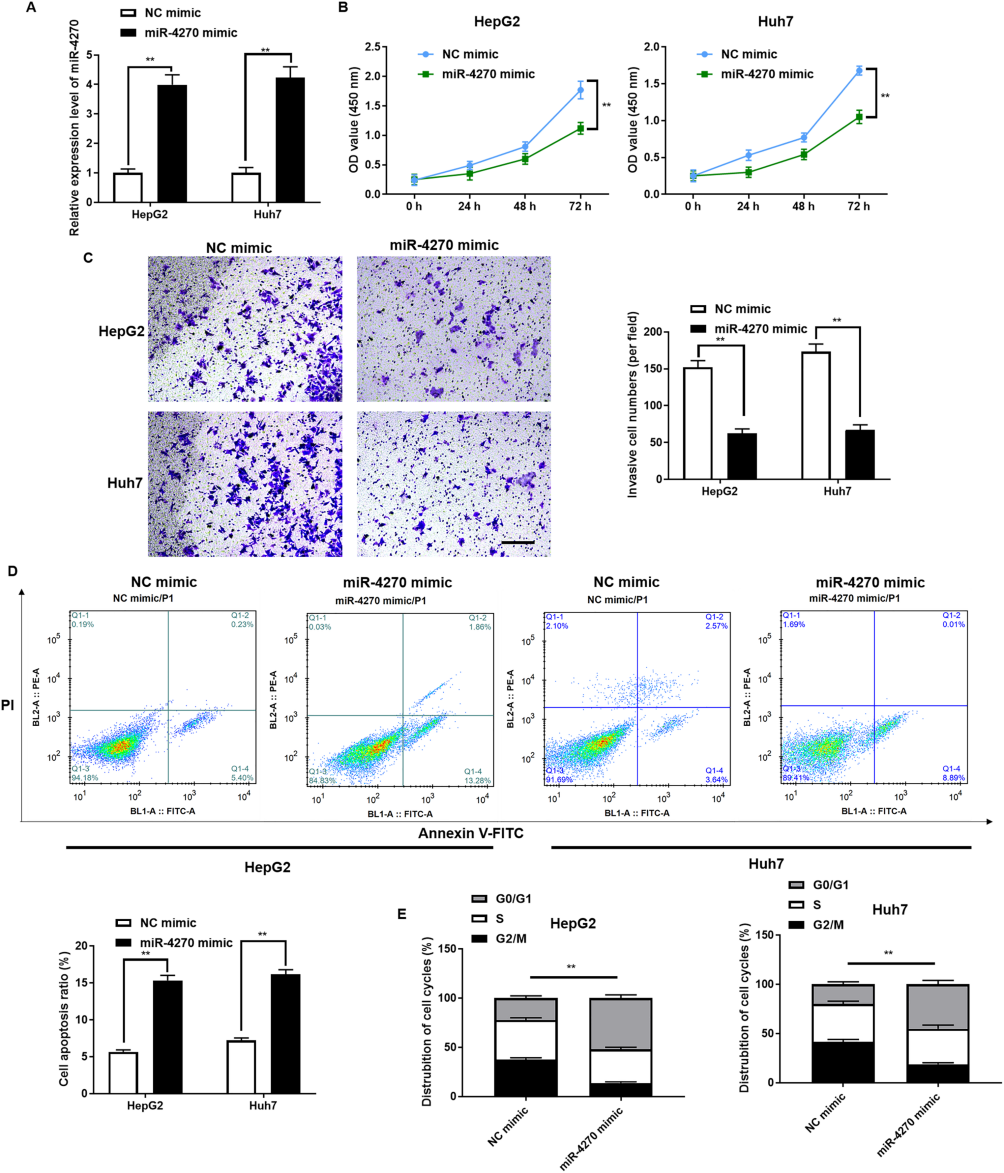
miR-4270 suppresses malignant characteristics in HCC cells. (A) RT-qPCR elucidation of miR-4270 levels in miR-4270 mimic transfected HCC cells, *t*-test. (B) miR-4270 mimic transfected HCC cells’ ability to proliferate was assessed *via* the CCK-8 assay, *t*-test and two-way ANOVA. (C) Transwell analysis evaluated the miR-4270 mimic transfected HCC cells’ invasion capability. Scale bar: 100 µm, *t*-test. (D) miR-4270 mimic transfected HCC cells was evaluated by Annexin V-FITC/PE assay, *t*-test. (E) Flow cytometry evaluation of the cycle distribution of miR-4270 mimic transfected HCC cells, *t*-test. **P < 0.01. *N* = 6.

To elucidate miR-4270’s biological function in HCC, HepG2, and Huh7 cells were transfected with the miR-4270 mimic, achieving transient overexpression. Figure 2A demonstrates the efficiency of miR-4270 mimic overexpression. miR-4270 overexpression markedly reduced both growth and invasion in HepG2 and Huh7 cells and induced apoptosis (Figs. 2B and 2D). Furthermore, miR-4270 overexpression substantially increased the number of cells in the G0/G1 phase while significantly decreasing those in the G2/M phase (Fig. 2E).

### miR-4270 targets the *DNMT3A* 3’UTR

**Figure 3:**
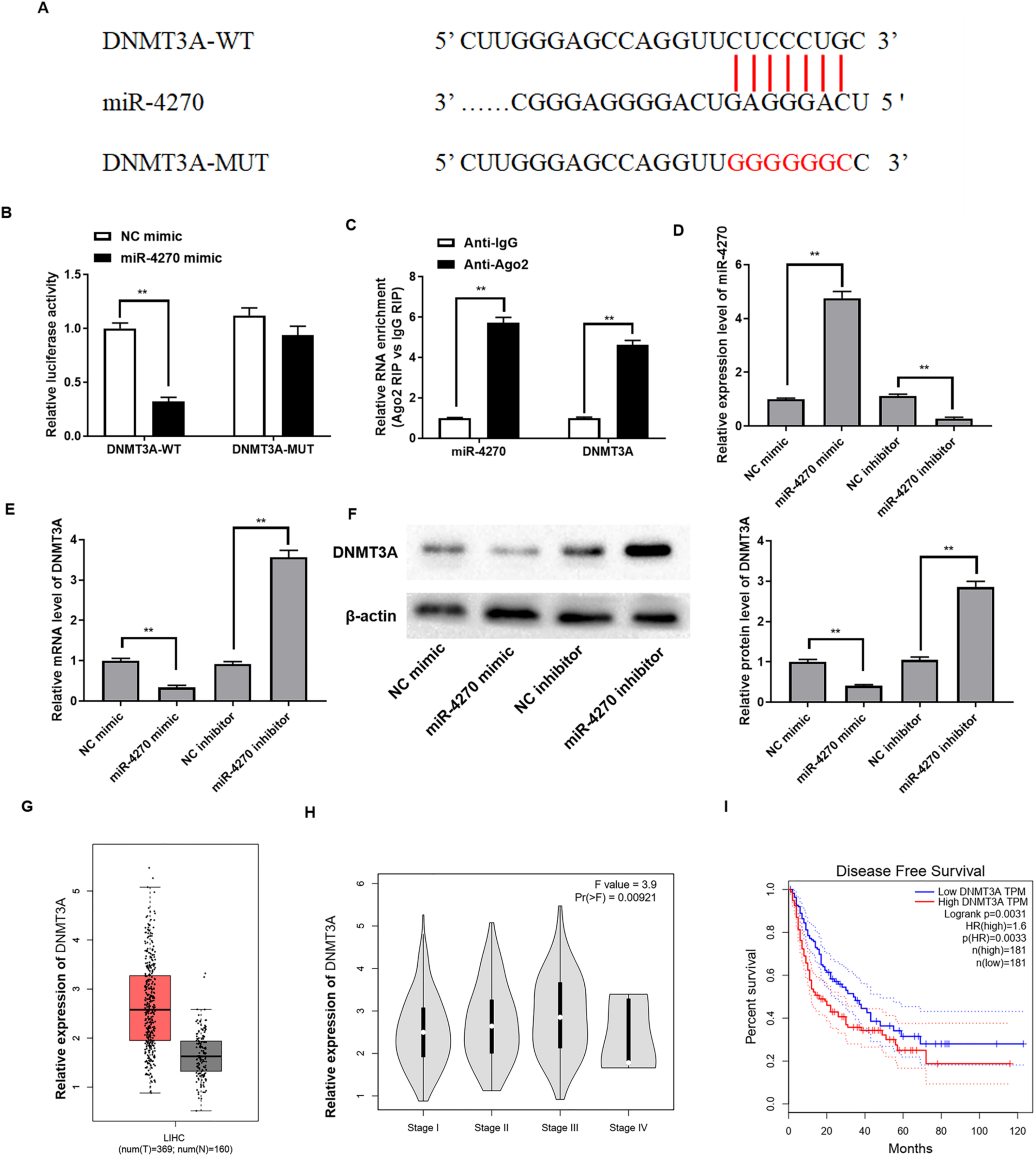
miR-4270 directly targets the 3′UTR of *DNMT3A*. (A) Binding sequences of miR-4270 and the 3′UTR of *DNMT3A*. (B) Relative luciferase activities of miR-4270 and *DNMT3A* were tested by luciferase reporter gene assay, *t*-test. (C) The RIP test was conducted to assess the relative enrichment level of miR-4270 and *DNMT3A* in HCC cells, *t*-test. (D) The mRNA levels of miR-4270, and (E) *DNMT3A* in miR-4270 mimic or miR-4270 inhibitor transfected HCC cells were assessed by RT-qPCR, *t*-test. (F) Western blot evaluation of *DNMT3A* levels in miR-4270 mimic or miR-4270 inhibitor transfected HCC cells, *t*-test. (G) Online analysis of *DNMT3A* levels in HCC tissues through the GEPIA website. (H) Online analysis of *DNMT3A* in different clinical stages of HCC patients through the GEPIA website. (I) Online analysis of disease-free survival curves of *DNMT3A* patients through the GEPIA website. **P < 0.01. *N* = 6.

*DNMT3A* was identified as a potential target gene of miR-4270 through StarBase online database analysis. Figure 3A illustrates the binding site of miR-4270 on the 3′UTR of *DNMT3A*. In addition, we further verified the binding relationship by dual-luciferase reporter and Ago2-RIP assays. The results indicated that binding of the miR-4270 mimic to *DNMT3A*-WT resulted in significantly less luciferase activity than the *DNMT3A*-MUT (Fig. 3B). Furthermore, the RNA enrichment of Ago2 on miR-4270 and *DNMT3A* was increased compared with IgG (Fig. 3C).

Subsequently, to explore whether miR-4270 could regulate *DNMT3A* expression, the miR-4270 mimic, and miR-4270 inhibitor were individually transfected into HepG2 cells. Figure 3D shows the interference efficiency of the miR-4270 inhibitor. *DNMT3A* protein and mRNA levels were markedly reduced by miR-4270 upregulation and increased by miR-4270 knockdown (Figs. 3E and 3F). Interestingly, we discovered that *DNMT3A* levels were substantially elevated in HCC patients compared to controls, shown by Sanger prediction, and were correlated with HCC clinical stage (Figs. 3G and 3H). Additionally, patients with elevated *DNMT3A* expression had notably poorer disease-free survival than those with low *DNMT3A* levels (Fig. 3I).

### miR-4270 inhibits *DNMT3A*-mediated *HGFAC* promoter methylation

**Figure 4:**
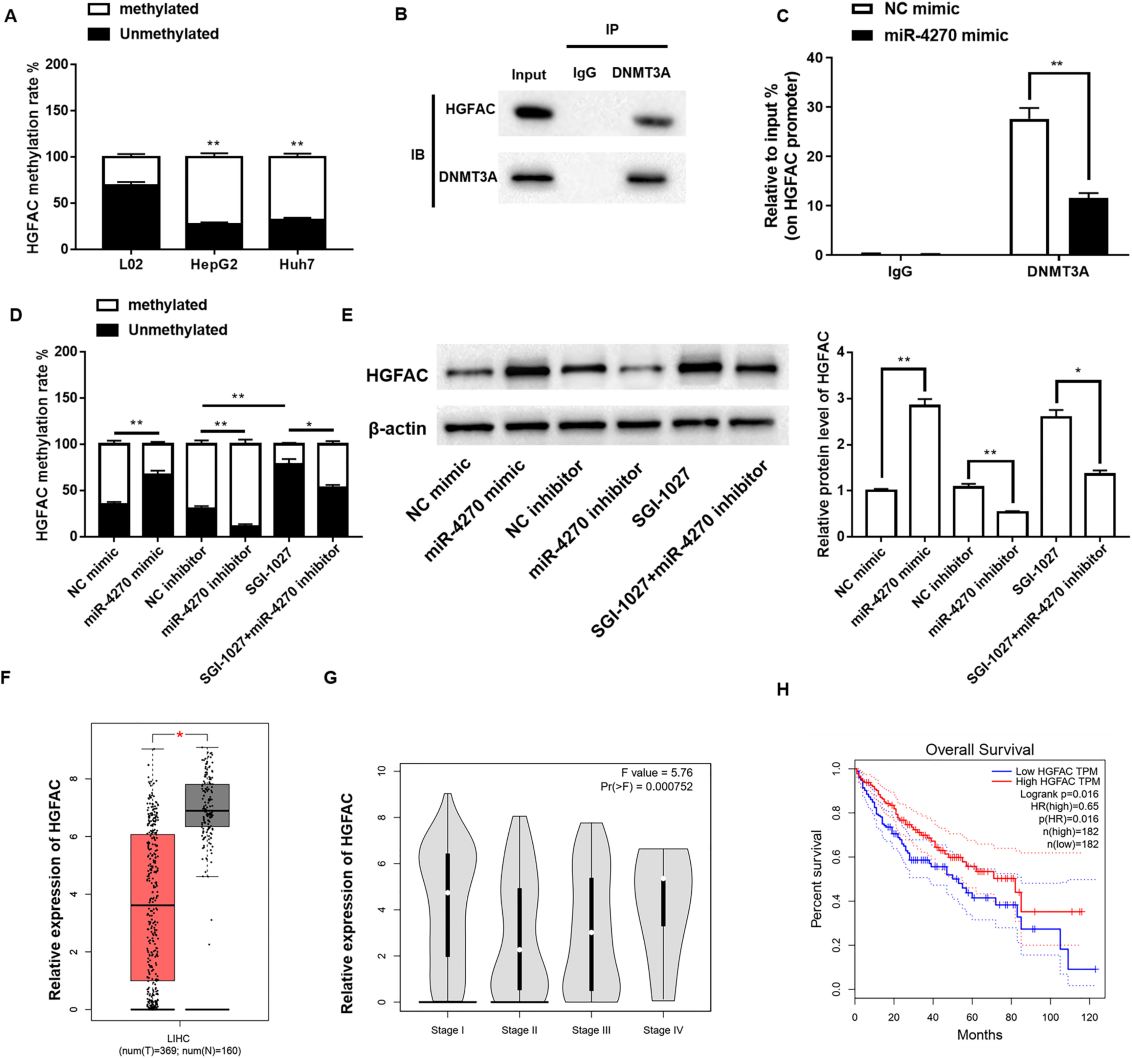
miR-4270 mitigates *DNMT3A*-induced methylation of the *HGFAC* promoter. (A) Methylation-specific PCR was employed to detect *HGFAC* methylation rate in L02, HepG2, and Huh7 cells, *t*-test and one-way ANOVA. (B) Co-IP evaluation of the protein interaction of *HGFAC* and *DNMT3A*. (C) The enrichment of *DNMT3A* in the *HGFAC* promoter region was elucidated by te ChIP assay, *t*-test. (D) Methylation-specific PCR was employed to detect *HGFAC* methylation rate in HepG2 cells transfected with miR-4270 mimic, miR-4270 inhibitor, or treated with SGI-1027, *t*-test and one-way ANOVA. (E) Western blot assessment of *HGFAC* levels in miR-4270 mimic and miR-4270 inhibitor transfected or SGI-1027 treated HepG2 cells, *t*-test and one-way ANOVA. SGI-1027: *DNMT3A* inhibitor (8 µM). (F) Online analysis of *HGFAC* levels in HCC tissues through the GEPIA website. (G) Online analysis of *DNMT3A* in different clinical stages of HCC patients through the GEPIA website. (H) Online analysis of disease-free survival curves of *DNMT3A* patients through the GEPIA website. *P < 0.05, **P < 0.01. *N* = 6. For image analysis, an inverted fluorescence microscope (#IX53; Olympus Corp, Tokyo, Japan) was utilized and Image-ProPlus 5.1 Chinese was employed for capturing images.

Previous studies have indicated a negative correlation between hepatocyte growth factor activator (*HGFAC*) expression and methylation of its promoter region, implying that DNA methylation may control *HGFAC* expression. Accordingly, we further analyzed whether *HGFAC* is regulated by DNMT3A methylation. Using the MSP, a significant increase was observed in *HGFAC* promoter methylation levels in HCC cells (Fig. 4A). Furthermore, Co-IP and ChIP assays demonstrated that DNMT3A could bind to *HGFAC* and was significantly enriched in its promoter region (Figs. 4B and 4C). Additionally, the methylation levels of the *HGFAC* promoter decreased significantly following miR-4270 overexpression and increased after miR-4270 inhibition. However, treatment with the DNMT3A inhibitor SGI-1027 markedly reversed the repressive effect of miR-4270 inhibition on *HGFAC* promoter methylation levels (Fig. 4D). Moreover, miR-4270 upregulation increased *HGFAC* protein levels, a trend reversed by miR-4270 downregulation of *HGFAC* and SGI-1027 treatment (Fig. 4E). Subsequently, we found that the *HGFAC* levels were significantly lower in HCC patients compared to controls, with Sanger prediction, and were correlated with the HCC clinical stage (Figs. 4F and 4G). Furthermore, patients with low *HGFAC* expression had significantly poorer disease-free survival than those with high *HGFAC* expression (Fig. 4H).

### *HGFAC* overexpression antagonizes the promotion of malignancy by the miR-4270 inhibitor

**Figure 5:**
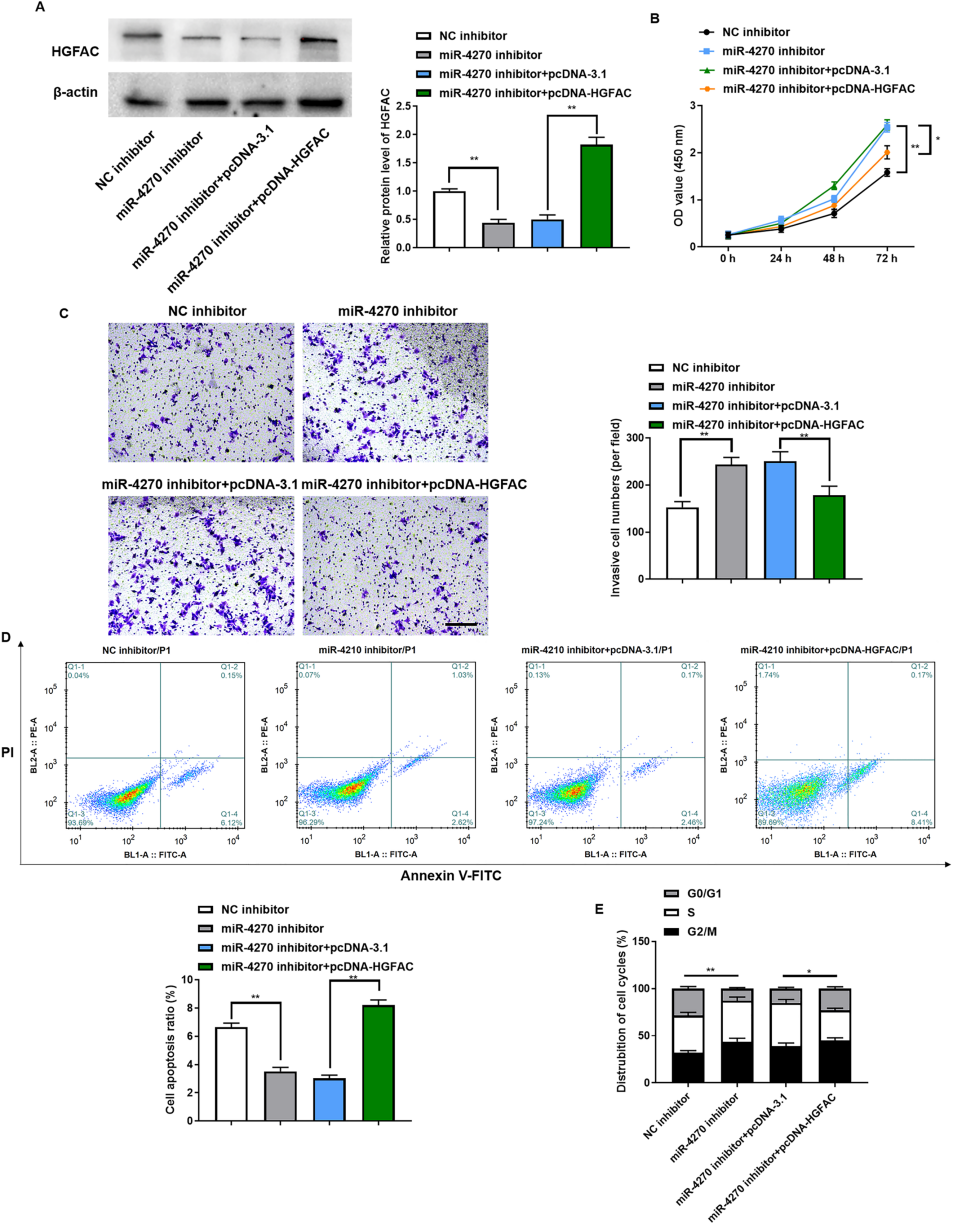
*HGFAC* overexpression could antagonize the facilitative effect of miR-4270 inhibitor on the malignant behavior of HCC cells. (A) Western blot assessment of *HGFAC* levels in HepG2 cells transfected with miR-4270 inhibitor or/and pcDNA-*HGFAC*, *t*-test. (B) Proliferation of miR-4270 inhibitor or/and pcDNA-*HGFAC* transfected HCC cells by CCK-8 assay, *t*-test and two-way ANOVA. (C) The invasion ability of miR-4270 inhibitor or/and pcDNA-*HGFAC* transfected HCC cells was assessed by transwell assay. Scale bar: 100 µm, *t*-test. (D) Annexin V-FITC/PE assay evaluation of the apoptotic ratio of HCC cells transfected with miR-4270 inhibitor or/and pcDNA-*HGFAC*, *t*-test. (E) Flow cytometry evaluation of the cycle distribution of miR-4270 inhibitor or/and pcDNA-*HGFAC* transfected HCC cells *t*-test. *P < 0.05, **P < 0.01. *N* = 6.

To determine if miR-4270 mediates its tumor-suppressive role in HCC cells through *HGFAC* regulation, we conducted rescue experiments involving co-transfection with the miR-4270 inhibitor and pcDNA-*HGFAC* plasmid. The efficiency of pcDNA-*HGFAC* transfection in HepG2 cells was verified by Western blotting (Fig. 5A). Transfection with pcDNA-*HGFAC* reversed the promotion of cell proliferation by the miR-4270 inhibitor (Fig. 5B), as well as invasion (Fig. 5C) and its inhibition of apoptosis (Fig. 5D). Moreover, *HGFAC* upregulation counteracted the miR-4270 inhibitor’s effect on the cell cycle, leading to an increased percentage of cells in the G0/G1 phase (Fig. 5E). Collectively, these data demonstrate that *HGFAC* inhibits HCC cell growth and serves an essential role in the pathway downstream of miR-4270.

### miR-4270 reduced xenograft tumor growth in mice

**Figure 6:**
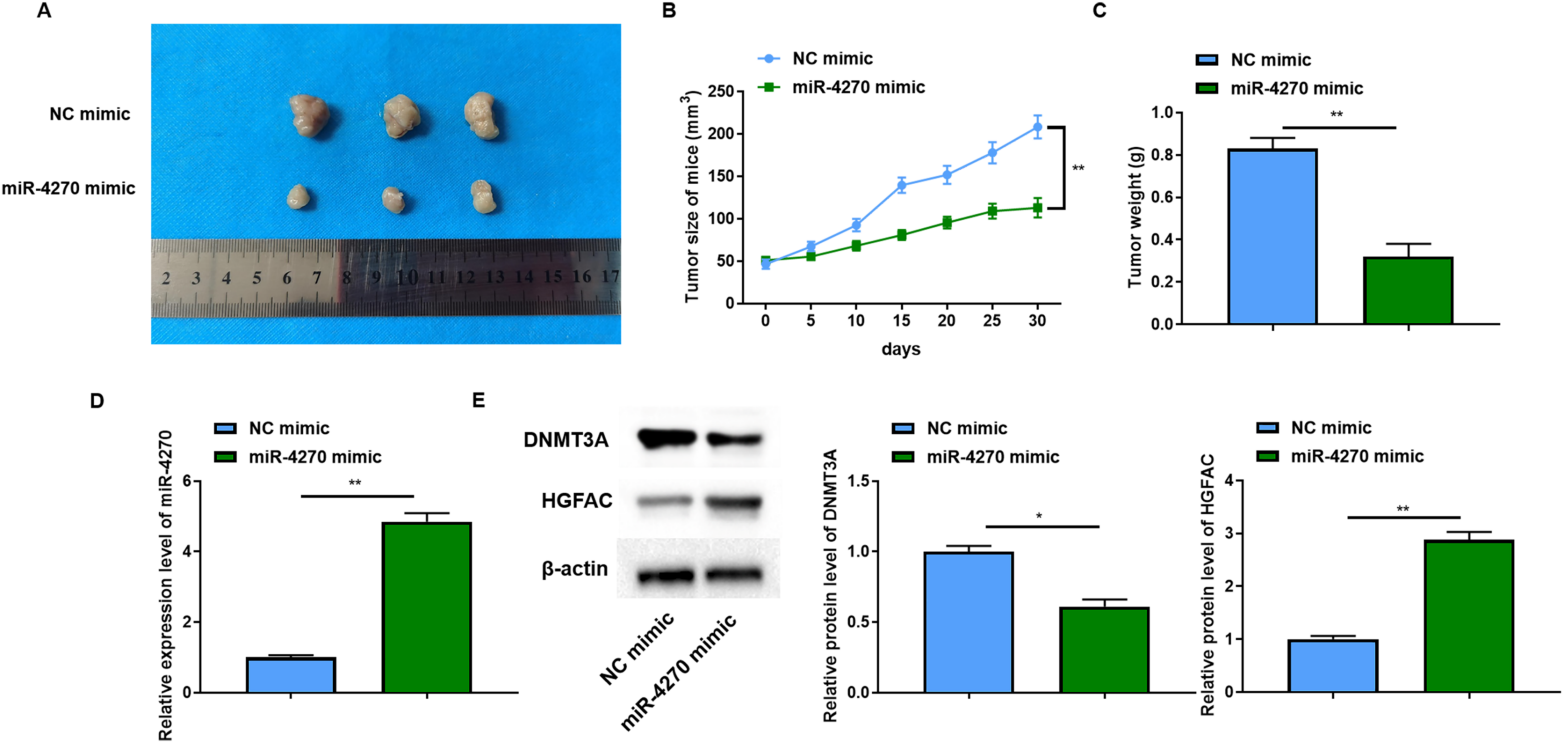
miR-4270 impeded xenograft tumor development in mice. (A) Representative images of each groups’ tumors, two-way ANOVA, and *t*-test. (B) Each groups’ tumor volumes, *t*-test. (C) Each groups’ tumor weight, *t*-test. (D) RT-qPCR evaluation of miR-4270 expression in each group of tumors, *t*-test. (E) Western blot evaluation of *DNMT3A* and *HGFAC* protein levels in each group of tumors, *t*-test. *P < 0.05, **P < 0.01. *N* = 12.

Following the initial focus of the *in vitro* tumor-suppressive effects of miR-4270, *in vivo* experiments were then performed. The results revealed a significant reduction in the size of the xenograft tumors in mice following miR-4270 overexpression (Fig. 6A). Measurements of tumor volume and mass also showed that miR-4270 overexpression retarded tumor growth in mice (Figs. 6B and 6C). Consistent with the *in vitro* findings, HGFAC protein levels increased significantly, and DNMT3A levels were decreased in tumor tissues from nude mice with increased miR-4270 expression (Figs. 6D and 6E). Taken together, these results confirmed that the upregulation of miR-4270 hindered the progression of HCC *in vivo*.

## Discussion

HCC is a global health problem and is one of the most common malignancies, ranking second in the causes of death due to cancer (Jiří, Igor & Mba, 2020; Elms, Badami & Dhanarajan, 2022). Given HCC’s high rate of metastasis and postoperative recurrence, understanding the molecular mechanisms underlying HCC initiation and progression is crucial, necessitating an in-depth search for key markers and gene targets pertinent to hepatocarcinogenesis and metastasis (Nagy et al., 2018; Qadir & Rizvi, 2017). Beyond common etiological factors such as hepatitis virus infection, cirrhosis, alcohol abuse, and fatty liver disease, genomic instability, and mutations also contribute significantly to the HCC predisposition. However, the precise pathological mechanism of HCC has not been fully elucidated.

Numerous studies on miRNAs have demonstrated their critical roles in regulating tumor cell proliferation, apoptosis, the cell cycle, and angiogenesis, processes of immense clinical significance in tumor development and progression (Hill & Tran, 2021; He et al., 2020). Current literature on miR-4270, although limited, indicates that its expression varies across various cancers, suggesting tissue-specific roles. Downregulation of its expression is associated with the progression of many tumors, and the mechanism may be to participate in gene regulation as a tumor suppressor (Wang et al., 2020). Wang et al. (2022a) reported reduced miR-4270 levels in nasopharyngeal cancer cell lines, associating its downregulation with increased cellular radiosensitivity. Additionally, reduced expression of miR-4270 in lung cancer tissues and cell lines underscores its potential role as a tumor suppressor (Zou et al., 2019). Sartorius et al. (2018) found that the levels of 10 miRNAs in HCC tissues were lower than those in matched non-tumor tissues, including miR-4270. This study used bioinformatics and tissue microarray data, providing evidence that miR-4270 levels are significantly downregulated in HCC tissues. Further, the miR-4270 mimic significantly reduced HCC cell proliferation and invasion and promoted cell apoptosis, compared with the NC mimic group.

MiRNAs play indispensable roles in HCC development, predominantly through their regulation of downstream target genes (Papanicolau-Sengos & Aldape, 2022). Based on the understanding that miR-4270 is a tumor suppressor in HCC, our study aimed to elucidate its underlying mechanisms by identifying downstream targets influenced by miR-4270. *DNMT3A* was found to be a target of miR-4270 by Sanger prediction as well as by dual-luciferase reporter assay and RIP assay verification. One of the early manifestations of many tumors is increased methylation of tumor suppressor genes, suggesting that changes in DNA methylation patterns may be among the first detectable tumor-specific changes associated with tumorigenesis (Hernandez-Meza et al., 2021).

Epigenetic alterations and modifications are important components of tumor initiation and progression, with DNA methylation being the most studied (Zhang et al., 2016). Previous studies have observed a correlation between altered gene methylation patterns and poorer prognoses, underscoring the significant role of epigenetic modifications in tumor development (Zhu et al., 2018). Recently reported differential methylation of promoters associated with the activation of oncogenes and premetastatic genes has been revealed to play a crucial role in cancer initiation and metastasis (Nishiyama & Nakanishi, 2021). DNA methylation, characterized by the addition of a methyl group to adenine or cytosine nucleotides within DNA sequences, is catalyzed by DNA methyltransferases (DNMTs) and constitutes a fundamental epigenetic imprint (Bestor & Verdine, 1994). In DNA methylation, s-adenosylmethionine serves as a methyl donor, facilitating a reaction catalyzed by three distinct DNMTs, namely, DNMT1, DNMT3A, and DNMT3B (Tajima et al., 2016). Among these, DNMT3A and DNMT3B are *de novo* methyltransferases that recognize methylated, hemimethylated, and unmethylated DNA (Okano et al., 1999). DNMT3A can introduce a methyl group into the demethylated CpG site to remethylate, regulating gene expression (Zhang et al., 2018). *DNMT3A* has been verified to be significantly up-regulated in HCC (Zhao et al., 2010). A study found that lncRNA SNHG5 decreased *DNMT3A* expression to suppress the methylation level of *SPATS2* in HCC (Yan et al., 2022a). This study demonstrated that miR-4270 effectively repressed DNMT3A-mediated methylation of the *HGFAC* promoter.

*HGFAC* is a novel serine protease that activates the precursor of hepatocyte growth factor and promotes angiogenesis, tumorigenesis, and regeneration in the tumor microenvironment (Fukushima et al., 2018). Three endogenous protease inhibitors of HGFAC, HAI-1, HAI-2, and protein C, regulate HGFAC activity. Notably, Guerra et al. (2019) initially reported the critical role of HAI-1 and HAI-2 as *HGFAC* repressors in gastric cancer, highlighting their significance in tumor progression. Research has indicated a correlation between reduced *HGFAC* levels in HCC and poor survival outcomes, with DNA hypermethylation contributing to this reduction in *HGFAC* expression, thereby proposing *HGFAC* as a prognostic biomarker for HCC patients (Yin et al., 2019). Similarly, we discovered that *HGFAC* promoter methylation levels were markedly increased in HCC cells, which were counteracted by miR-4270 upregulation. In contrast, downregulation of miR-4270 suppressed HGFAC protein expression and increased methylation of the *HGFAC* promoter, effects that were counteracted by treatment with SGI-1027. Furthermore, *HGFAC* overexpression antagonized the promotion of malignant behavior by the miR-4270 inhibitor in HCC cells.

In conclusion, this study, by *in vivo* and *in vitro* analyses, revealed that miR-4270 can reduce growth, invasion, and migration in HCC cells. Mechanistically, miR-4270 inhibited the malignant behavior of HCC by suppressing DNMT3A-mediated methylation of the *HGFAC* promoter. Nevertheless, a more comprehensive mechanism of miR-4270 in HCC cells still needs further investigation. A limitation of our study was the omission of control cells and mice from the experimental design, preventing us from ruling out potential effects attributable to the NC mimic or NC inhibitor treatments. Moreover, our findings indicate a relative under-expression of miR-4270 in HCC cells. However, determining whether similar patterns of miR-4270 expression occur in tumor tissues and sera of HCC patients requires verification through comprehensive clinical trials.

## Supplemental Information

10.7717/peerj.16566/supp-1 Supplemental Information 1 PRISMA 2009 Checklist.

10.7717/peerj.16566/supp-2 Supplemental Information 2 Raw Data.

10.7717/peerj.16566/supp-3 Supplemental Information 3 Raw images of the proteins in Figures 3 and 4.

10.7717/peerj.16566/supp-4 Supplemental Information 4 Raw images of the proteins in Figures 5 and 6.

10.7717/peerj.16566/supp-5 Supplemental Information 5 MIQE Checklist.

## Additional Information and Declarations

### Data Availability

The following information was supplied regarding data availability: The raw data are available in the Supplemental Files.
