# Supplementary figures and images for: miR-4270 suppresses hepatocellular carcinoma progression by inhibiting DNMT3A-mediated methylation of HGFAC promoter
Source: PeerJ. 2023 Dec 5;11:e16566. doi: 10.7717/peerj.16566 (PMC10704985; doi:10.7717/peerj.16566)

Figure 3F

DNMT3A


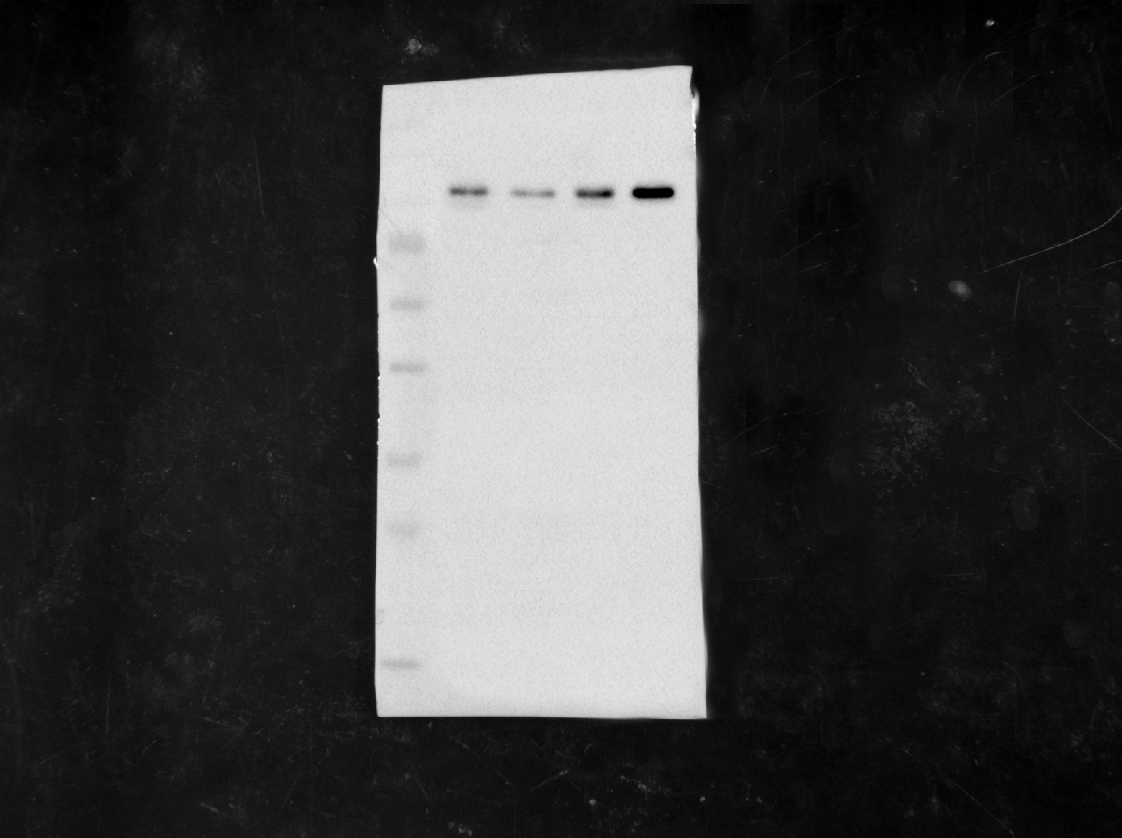


β-actin


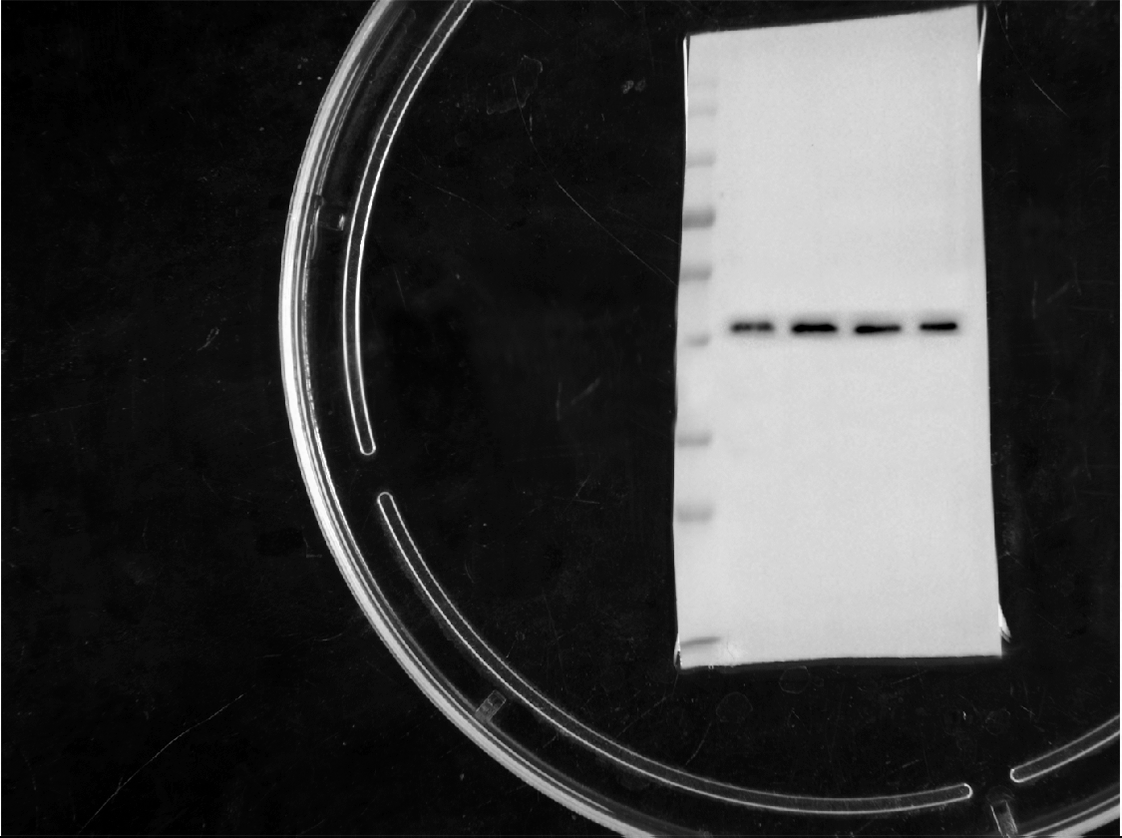


Figure 4B

HGFAC


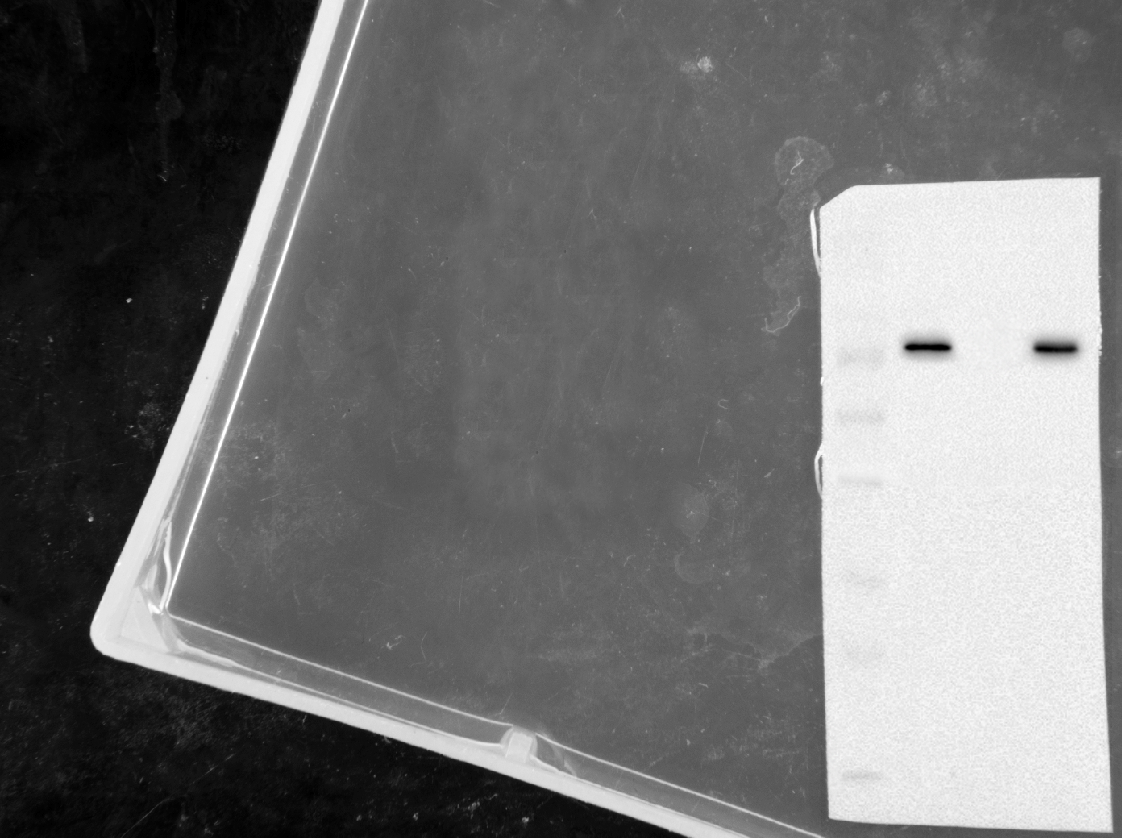


**DNMT3A**


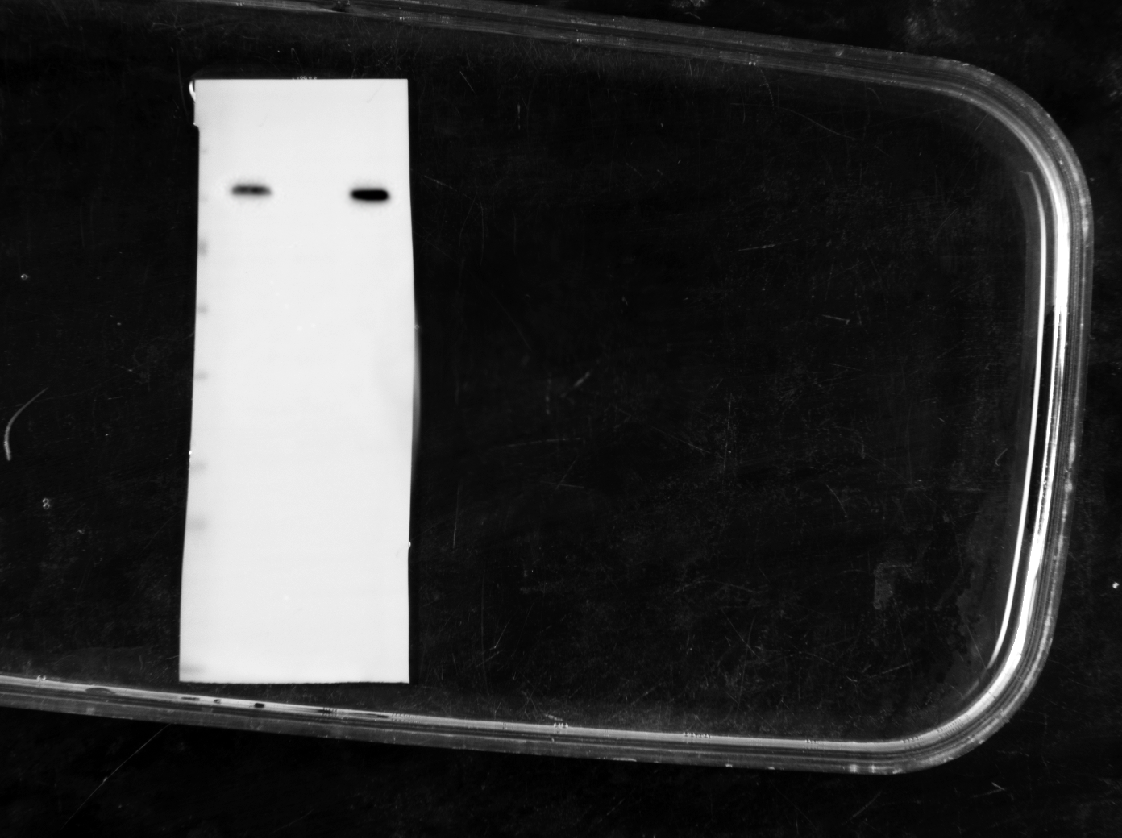


Figure 4E

**HGFAC**


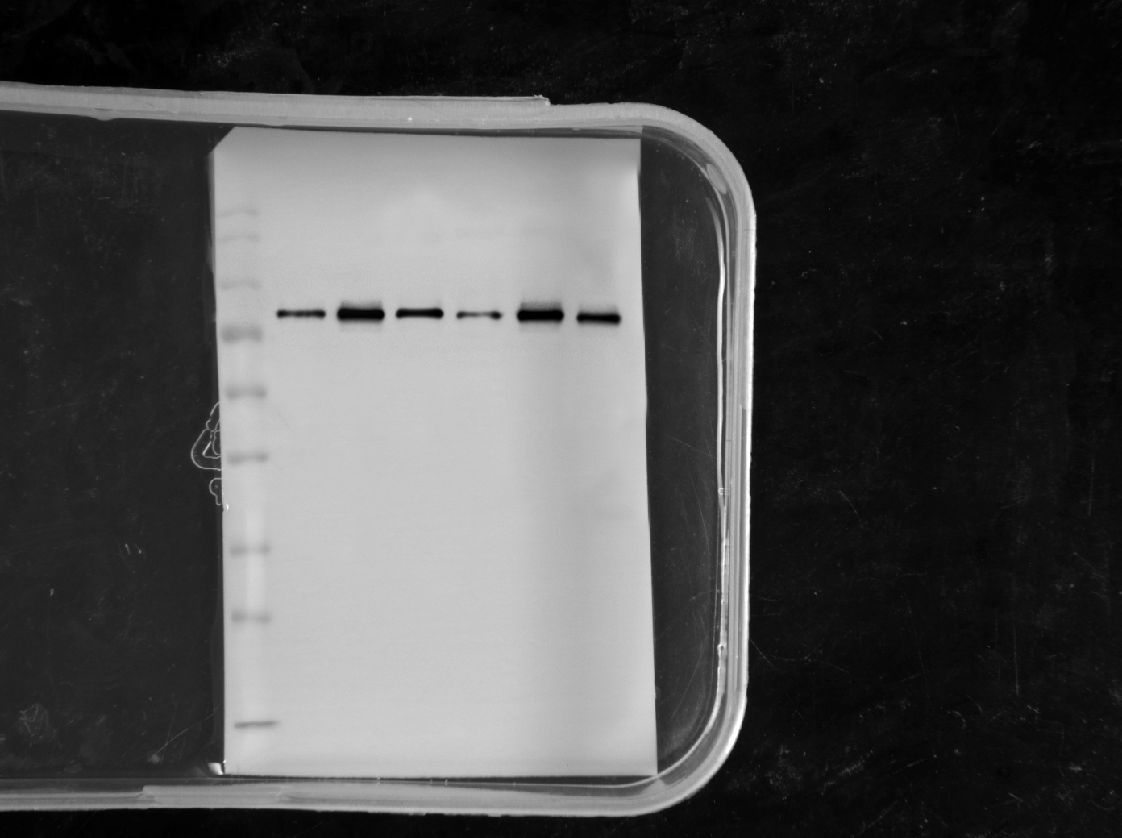


**β-actin**


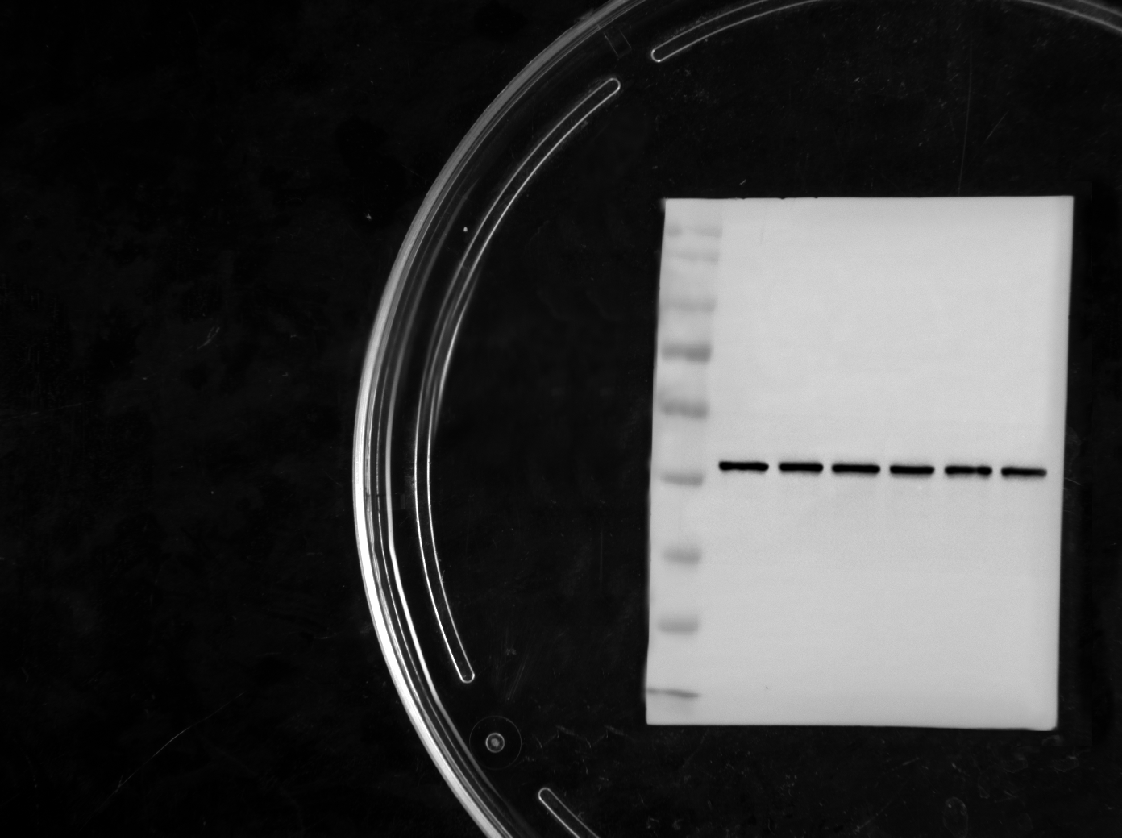

Supplement: Supplemental Information 3 [file peerj-11-16566-s003.docx]

Figure 5A

**HGFAC**


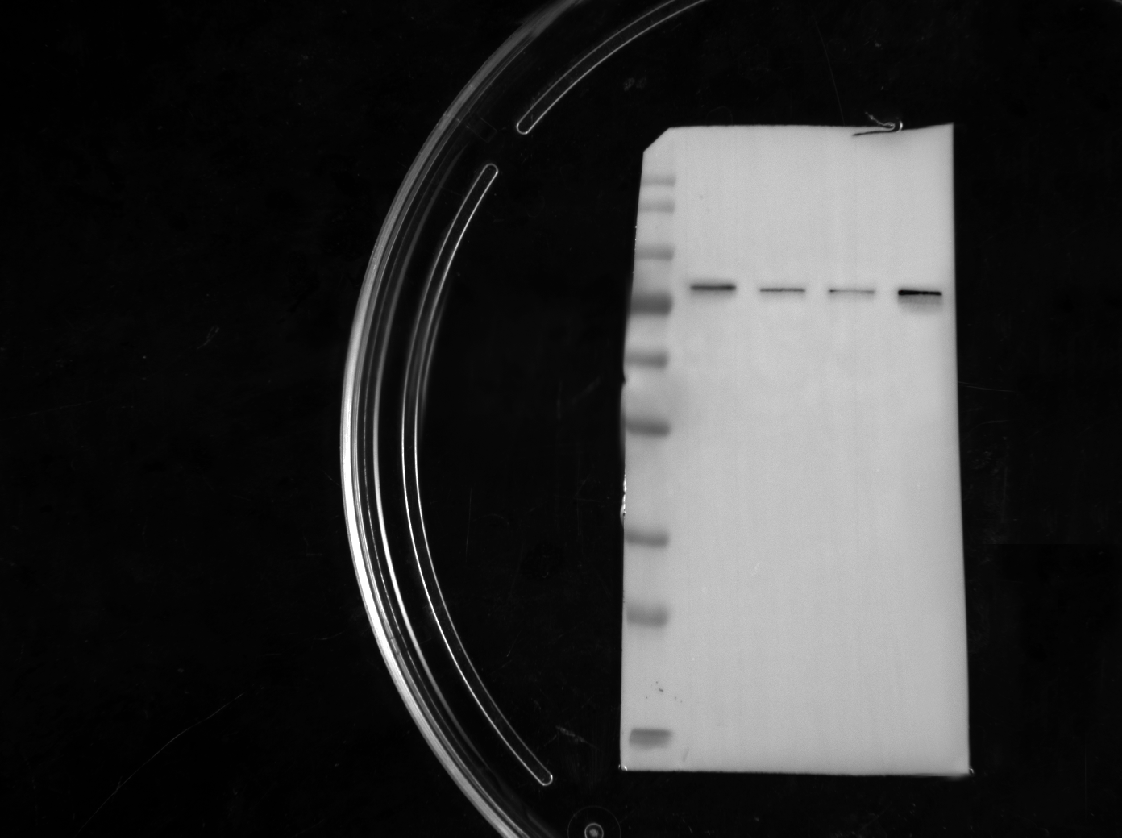


**β-actin**


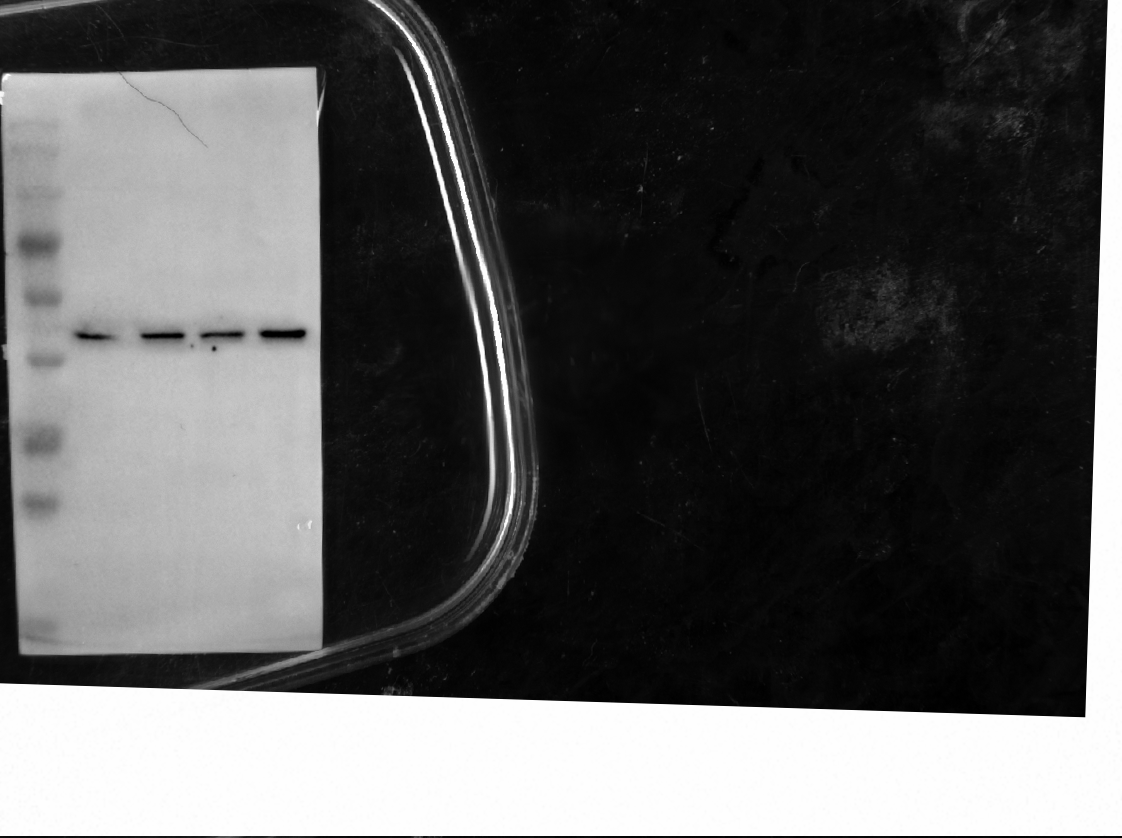


Figure 6E

**DNMT3A**


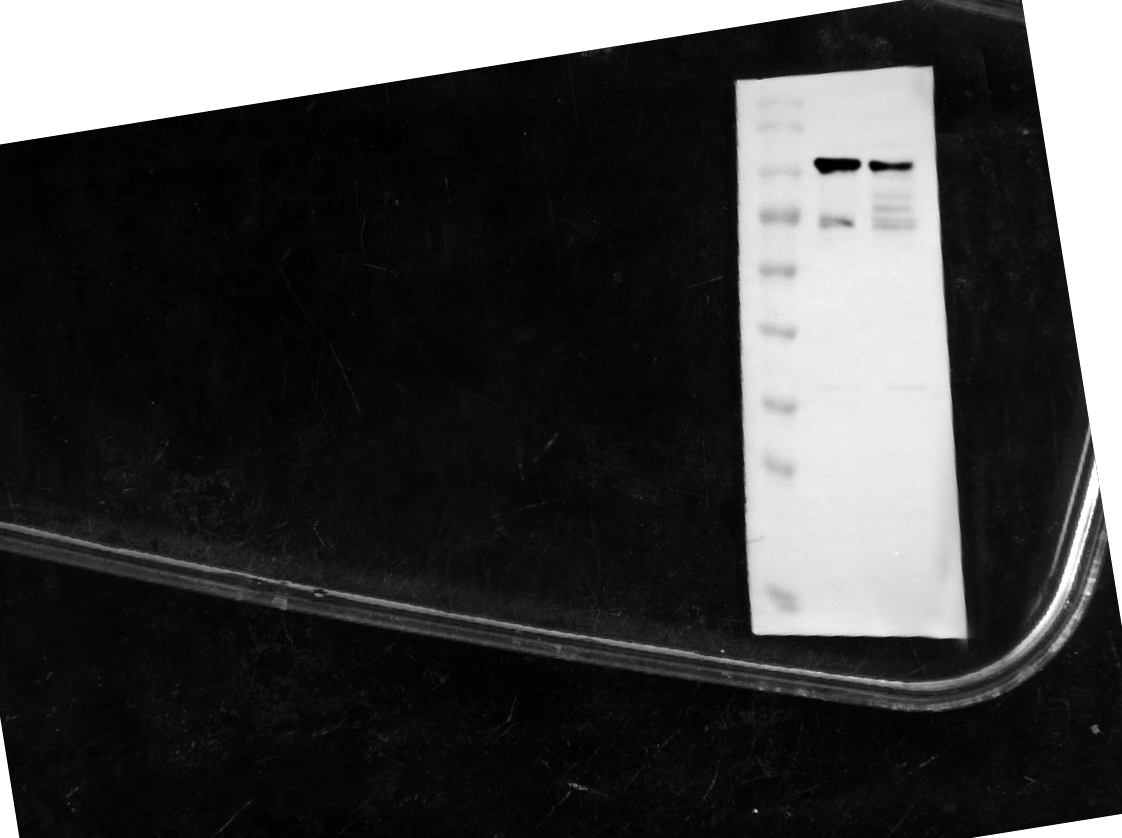


**HGFAC**


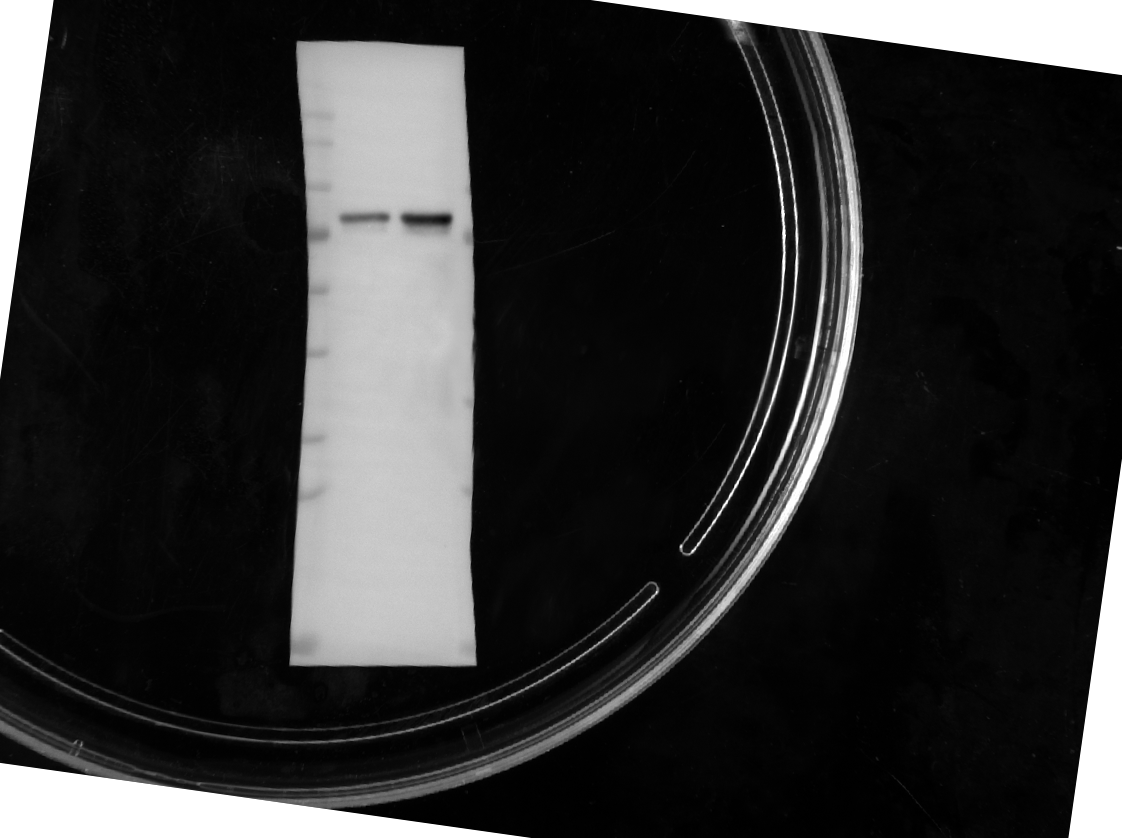


**β-actin**


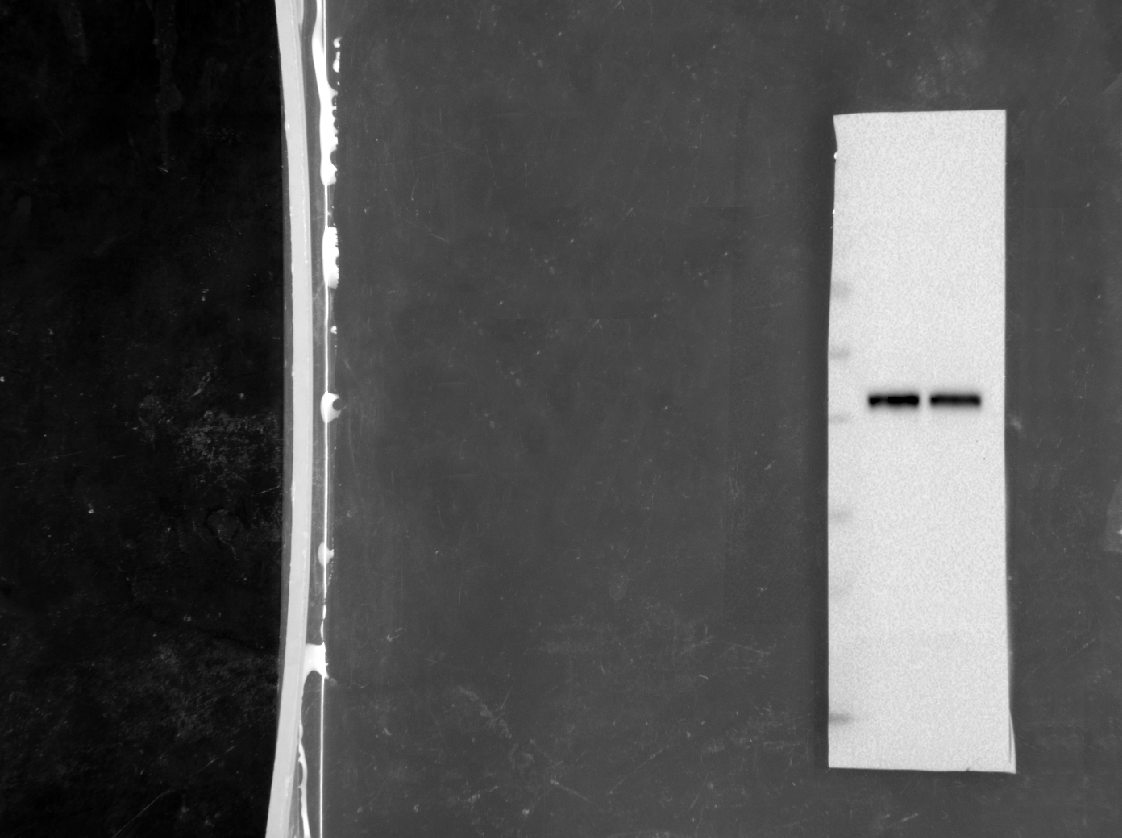

Supplement: Supplemental Information 4 [file peerj-11-16566-s004.docx]
